# Ave Atque Vale

**Published:** 1970-01

**Authors:** 


### Ave Atque Vale

### Some Reflections by the Retiring Hon. Treasurer, Bristol Medico-Chirurgical Society

As the years pass an Honorary Treasurer grows an oyster of experience large enough to nourish a few pearls out of the grit of time. A record of these might be an interesting amusement to the members of the Society: perhaps it might be a help and a warning to future treasurers.

The position is entirely Honorary: years of anxious study has proved that there is no dividend for the Treasurer and that embezzlement is but a vulgar dream. Appointment to the post starts in the comfortable military tradition of—"you have volunteered". Unopposed re-appointment every year makes happy nonsense of the annual nature of the work. Tenure extends indefinitely to the contented murmur of the members attending successive General Meetings.

The post costs the Treasurer a few unaccounted shillings on petrol, telephone, stamps and general wear and tear: statistics show that Hon. Treasurers are spectacularly liable to "the stroke", although the same researchers fortunately record a remarkable recovery rate among the emeriti, retired.

It has been noted that the Hon. Secretary probably spends unaccounted pounds compared with the shillings of the Hon. Treasurer and all members should be aware of the inordinate, if quite voluntary cost of being President of the "Med-Chi." The Senior Common Room presents bills which the Hon. Treasurer does not handle, but he is constrained to wonder about them from time to time. This may explain to the mercenary Caledonian mind why there have been sixteen Presidents, three Secretaries and one Treasurer during the past sixteen years. But such ruminations are indiscreet, if not frankly irreverent.

The executive positions in our Society are not only Honorary but Honourable, too. For nearly a century willing members have been found happy to fill vacancies and the Society has flourished with an unabated success not commonplace in other parts of the Realm. We are a Learned Society which has kept its spirit, dignity and aspirations through a long history: which has moved with the times, gently introducing a greater range of subject and speaker beyond the strict limits of professional medicine: which has maintained traditional quality by fine audience participation, so as to delight and reward the industry of our successive speakers. It is the grand attendance record of our ordinary members which makes our Society an important community and builds the Honourable dignity investing all the executive positions and makes them so well worth while.

Honorary and Honourable though he be, the Treasurer, like his colleagues, has work to do: much of it is dull routine, but of a bulk only manageable with the help of an able, willing and interested secretary. Society members may not always be aware of the immense debt owed to the anonymous assistance of kindly folk like the Hon. Treasurer's careful and conscientious secretary.

Perhaps because the crude but essential pillars of our Learned Society are of "brass", financial affairs have little general appeal. However, in recent years two important matters of policy have been accepted; the subscription was increased from one and a half guineas to two guineas—the first increase in living memory but made imperative by the soaring printing costs of our Journal: a goodly majority of members have agreed to pay the annual subscription by Bank Order, thus saving untold labour, stamps and stationery otherwise expended on yearly demands, reminders and re-reminders involving a Society over 500 strong and still growing.

It would be a boon if all members paid thus automatically, but, of course, one must assume that some indigent souls may not have bank accounts, or, more simply, feel that the Hon. Treasurer should be seen to work. A handful obviously like to wait for four or five years so that they can have the pleasure of signing a really worth while cheque to the Society. A very few like to thrust a handful of warm coins into an unsuspecting Treasurer's hand at a strikingly awkward moment, assuming recognition seldom reciprocated. Fortunately, the Treasurer knows that within a week the unknown member will write a reproachful letter claiming his receipt.

Cheques are not always what they purport to be; they may be signed or unsigned, dated, undated, post-dated or ante-dated—in one case, by a century! They are never blank. Signatures may be sadly indecipherable and on occasion the member's identity can be established only with difficulty using his cheque number and the kind offices of his bank manager. For many years one of our most lovable members paid one guinea, always forgetting to alter his bank order. On one dreadful occasion a subscription was demanded from one of our Honorary Members. When it was paid without murmur the gaffe was realized.

Members resign and come back again; they change addresses; the Journal is not delivered or too many arrive; the Treasurer's mail varies. Once a subscription was charged against a member's wife's account causing domestic suspicion beyond the value of one and a half guineas.

Society members with a fault to find write some wonderful letters; the more real the grievance the more righteous the letter. The Treasurer replies regularly in a "mea culpa" fashion and this seems to generate amity. A more serious problem has been trying to keep note of the deaths of members and, since some are missed, inevitably subscription demands have been received by bereaved relatives. Naturally this gives offence and generates some very stiff letters, but it is unfortunately liable to remain a human failing lying permanently at the Hon. Treasurer's door.

The year's work reaches its climax when "the books" go to the auditors in July: to be returned, at the earliest, two days before the Annual General Meeting in October as the Statement of Accounts, duly accredited. Thus, for a not inconsiderable fee, and by the exercise of specialist arithmetic, a simple credit and debit account is resolved into an incomprehensible statement to bemuse members and Treasurer alike.

Because our Society is older than Bristol University and was in some measure accoucheur to the Medical Faculty, many important benefits have accrued to the Society. We have free accommodation and splendid facilities for our meetings; we have great advantages and privileges in the Medical Library, not least the willing and invaluable labours of the Librarian; the bare cost of running this highly successful Society could probably be covered comfortably by a half guinea subscription. But we have a Journal, almost as venerable as the Society itself, and therefore possessing antique as well as intrinsic value. It is one of the very few provincial medical journals left: it would have sunk with the rest in a sea of rising costs had the Society not been sentimental and generous to a fault in its support.

Of each member's subscription, custom has decreed that sixteen shillings be allotted to pay for the quarterly Journal. This allocation amply sufficed as long as Caxton's innovation was reasonably cheap. During the past two decades, only for a few years did this money cover the printing costs and this was when we had an excellent but economical Editor who produced very few Journals. During this halcyon period the Journal account prospered. Since then we have had industrious editors, even two at a time, and sometimes one issue of the Journal swallows up the annual product of the many sixteen shillings!

The answer is, of course, a larger circulation and lots of financially juicy advertisements. The Journal committee has tried every permutation of the printing game: we even had a failed tave-over bid from a commercial gent (much recommended by an expensive management consultant), who promised to make the Journal pay a profit. When it did not, he ceased to answer the Treasurer's importunate letters and telephone calls, and the Journal still appears regularly thanks to generous transfers of money from the Society account.

The cost of printing would be unbelievable except that it increases every year. Publishing, despatch and advertising have become closed shops, very expensive, if not impossible for amateurs. Perhaps, apart from Editorial opinion, policy and material, the Journal would better be taken over by a large publishing firm. One such firm, with at least a kindly feeling towards our local medical tradition, has offered such a service.

The Journal is the child of the Society and we should keep it alive: soon, however, we shall have to decide its validity as well as its cost. Its survival so far owes much to the devotion of our medical librarian, but even willing horses retire and the loss of so much quiet, contented but sweated labour could be disastrous.

The Society, therefore, has one big Charity — the Journal: if the large contribution to this can be reduced by commercial management, then the Society could well be Patron to other Good Causes as a regular feature of its activities. We subscribe £50 each year to the Medical Library — another child of the Society, and, therefore, of natural, continuing cost to the parent. Sometimes, as fortuitous occasion arises, we have given money to other ends but there may be a need for more organised generosity, perhaps the appointment of an Almoner—Honorary and Honourable, of course. We could afford a respectable annual subscription now to any worthy medical cause: as only one suggestion, what more excellent, local and deserving one than the Jenner Trust. The West of England has produced two really great men: all Irishmen can be trusted to keep green the memory of the Englishman Patrick. Let the "Med-Chi. Soc." do something similar for the rather neglected late parish doctor of Berkeley, who saved more lives than the good St. Patrick could imagine to exist, even in his most evangelical dreams.

Being Hon. Treasurer may consist of filling large ledgers, checking bank statements, answering letters of reproach written in English, Spanish, Japanese, Greek and even Yankee, but it is somehow an enjoyable exercise, becoming in a little time an abrasive pleasure and, in the end, a simplicity of tolerance practised in routine. . . . Experientia docet . . . J.M.

